# Genome survey of Chinese fir (*Cunninghamia lanceolata*): Identification of genomic SSRs and demonstration of their utility in genetic diversity analysis

**DOI:** 10.1038/s41598-020-61611-0

**Published:** 2020-03-13

**Authors:** Erpei Lin, Hebi Zhuang, Jinjian Yu, Xueyu Liu, Huahong Huang, Muyuan Zhu, Zaikang Tong

**Affiliations:** 1 The State Key Laboratory of Subtropical Silviculture, Institute of Biotechnology, College of Forestry and Biotechnology, Zhejiang A & F University, Lin’an, Hangzhou, 311300 Zhejiang, China; 2 Key Laboratory for Cell and Gene Engineering of Zhejiang Province, Institute of Genetics, College of Life Sciences, Zhejiang University, Hangzhou, 310058 Zhejiang, China

**Keywords:** Genetic markers, DNA sequencing, Plant molecular biology

## Abstract

Chinese fir (*Cunninghamia lanceolata*) is an important coniferous species that accounts for 20–30% of the total commercial timber production in China. Though traditional breeding of Chinese fir has achieved remarkable success, molecular-assisted breeding has made little progress due to limited availability of genomic information. In this study, a survey of Chinese fir genome was performed using the Illumina HiSeq Xten sequencing platform. K-mer analysis indicated that Chinese fir has a large genome of approximately 11.6 Gb with 74.89% repetitive elements and is highly heterozygous. Meanwhile, its genome size was estimated to be 13.2 Gb using flow cytometry. A total of 778.02 Gb clean reads were assembled into 10,982,272 scaffolds with an N50 of 1.57 kb. In total, 362,193 SSR loci were detected with a frequency of 13.18 kb. Dinucleotide repeats were the most abundant (up to 73.6% of the total SSRs), followed by trinucleotide and tetranucleotide repeats. Forty-six polymorphic pairs were developed, and 298 alleles were successfully amplified from 199 Chinese fir clones. The average PIC value was 0.53, indicating that the identified genomic SSR (gSSR) markers have a high degree of polymorphism. In addition, these breeding resources were divided into three groups, and a limited gene flow existed among these inferred groups.

## Introduction

Chinese fir (*Cunninghamia lanceolata* (Lamb.) Hook), an evergreen conifer native to southern China and northern Vietnam, belongs to the Cupressaceae family. Because of its fast growth, desirable wood properties, and high resistant to diseases, it has been widely cultivated in China for over 3000 years. Chinese fir is a timber species that currently has the largest plantation area in China, and its annual harvest accounts for 20–30% of total commercial timber production^1^. The systematic breeding of Chinese fir, including provenance tests, cross-breeding and clonal selection, has been conducted since the 1960s. Many first-, second- and third-generation seed orchards have been established^2^. With the accumulation of breeding resources, the management of their genetic diversity and genetic relationships has become increasingly important.

Genetic diversity evaluation of germplasm resources can provide crucial reference information for germplasm conservation, selection of elite germplasms, and parental selection for crosses. In the last twenty years, molecular genetic diversity in Chinese fir has been evaluated many times. You *et al*. utilized random amplified polymorphic DNA (RAPD) technologies to analyse the genetic diversity of 7 representative provenance samples in China for the first time. They found that there was a relatively high level of genetic diversity among these provenances and deduced that the central-western part of Nanling Mountain is a central distribution zone of Chinese fir^3^. Subsequently, other molecular markers, including amplified fragment length polymorphism (AFLP)^4^, sequence-related amplified polymorphism (SRAP)^5^, and inter simple sequence repeats (ISSRs)^6^, were also adopted to analyse the molecular genetic diversity of Chinese fir. However, these dominant markers cannot distinguish between heterozygotes and homozygotes; thus, they are insufficient for a precise assessment of molecular genetic diversity in Chinese fir.

Microsatellites or simple sequence repeats (SSRs) are codominant markers and can be divided into expressed sequence tag-SSRs (EST-SSRs) and genomic SSRs (gSSRs). With the advantages of a high level of polymorphism, high specificity and repeatability, and extensive genomic coverage, SSR markers have been widely used to disclose genetic diversity and relationships in many crop species, such as rice (*Oryza sativa*), wheat (*Triticum aestivum*), soybean (*Glycine max*) and *Pennisetum* species^7–11^. Few studies on the identification and application of polymorphic SSR markers have been reported in Chinese fir. Using the Chinese fir EST sequences deposited in public databases, Zhang *et al*. first developed EST-SSR primers and evaluated the molecular genetic diversity of 30 clones^12^. Through the combination of SSR mining and multiplex-PCR methods, a flow chart of EST-SSR marker development was established using Chinese fir transcriptome data from next-generation sequencing (NGS). In addition, 28 polymorphic EST-SSR loci were obtained, which were verified as suitable for identifying the provenances, even individuals of Chinese fir^13^. A relatively high level of genetic diversity within different Chinese fir samples was also revealed using EST-SSR primer pairs in previous studies^14,15^. However, only 11 polymorphic gSSR loci of Chinese fir have been reported thus far, of which 7 derived from candidate genes involved in wood formation, and further experiments showed that the polymorphic information content (PIC) values from gSSR primers were higher than those from EST-SSR primers^16,17^. In addition, the Chinese fir genome is large, with most sequences being non-coding, and it exhibits abundant variations among different genotypes. Therefore, it is necessary to develop more polymorphic gSSR primers.

NGS has many advantages, including high throughout and rapid sequence data generation. Genome survey sequencing via NGS not only provides information on the genome structure of a species, such as genome size, heterozygosity and repeat contents, but also can generate a large amount of sequence data for the development of new gSSR markers. Recently, many high-quality gSSR markers have been developed in sesame (*Sesamum indicum*)^18^, buckwheat (*Fagopyrum tataricum*)^19^, pistachio (*Pistacia vera*)^20^ and elephant grass (*Pennisetum purpureum*)^21^ based on genome survey sequencing. However, until now, there have been no reports on the genome survey sequencing of Chinese fir.

In this study, we aim to (1) estimate the genome size, GC content, and heterozygosity of Chinese fir, (2) conduct a genome-wide identification of SSRs and develop novel gSSR primers, and (3) discover the genetic diversity and population structure of the Chinese fir germplasm. To our knowledge, this is the first report on the genome structure and genome-wide SSRs of Chinese fir. The results will provide valuable information for the whole genome sequencing of Chinese fir and contribute to accelerating the progress of genetic map construction and SSR marker-assisted breeding in this important timber tree.

## Results

### Genome size estimation by flow cytometry

**Figure 1:**
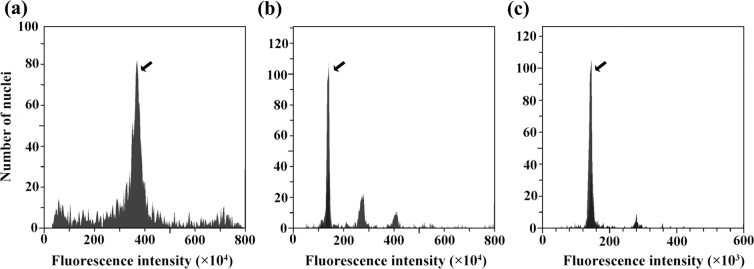
Estimation of genome size in Chinese fir by flow cytometry. G_0/1_ nuclei peaks (indicated by the arrows) were obtained after flow cytometric analysis of PI-stained nuclei suspensions prepared from leaf samples. Each sample was determined with 10 replicates, and the CV of each peak was set below 5%. (**a**) G_0/1_ nuclei peak of Chinese fir (2 C = 26.99 pg, fluorescence intensity 3.67 × 10^6^). (**b**) G_0/1_ nuclei peak of barley (2 C = 10.43 pg, fluorescence intensity 1.38 × 10^6^). (**c**) G_0/1_ nuclei peak of *P. trichocarpa* (2 C = 0.98 pg, fluorescence intensity 1.38 × 10^5^).

The nuclei of Chinese fir, barley (*Hordeum vulgare*), and *Populus trichocarpa* were stained with propidium iodide (PI). Fluorescent signals were captured using a flow cytometer. The cell peaks of three species are shown in Fig. 1. Most of the nuclei of Chinese fir leaves formed a large peak at 3.67 × 10^6^ (fluorescence intensity) corresponding to the G_0/1_ phase of the cell cycle (Fig. 1a). Histograms of barley and *P. trichocarpa* contained two main peaks that corresponded to G_0/1_ nuclei of both species (Fig. 1b,c). The genome sizes of barley and *P. trichocarpa* are 5.1 Gb and 480 Mb, respectively^22,23^. Therefore, the genome size of Chinese fir was calculated to be 13.20 ± 0.57 Gb.

### K-mer analysis

**Figure 2:**
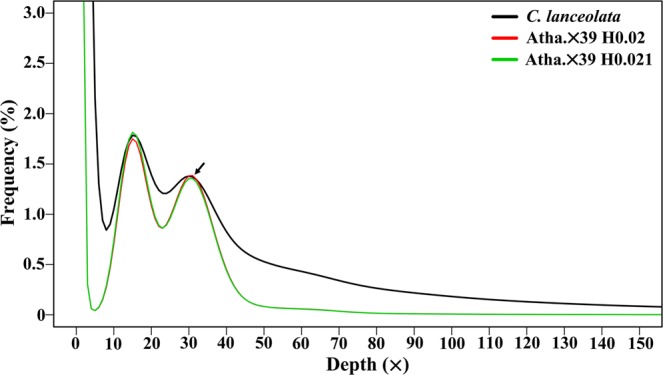
K-mer (K = 17) analysis for estimating the genome size of Chinese fir. The X-axis is depth (×) and the Y-axis is the proportion that represents the frequency at that depth divided by the total frequency of all depths. The peak indicated by the black arrow is the main peak corresponding to the expected K-mer depth. The genome size is estimated according to the formula: genome size = total K-mer number/peak depth (total K-mer number = 347,935,256,501). Atha. ×39 represents the depth of *Arabidopsis thaliana* is 39. H0.02 and H0.021 means that heterozygous rate is 2% and 2.1%, respectively. The peaks of Atha. ×39 were used as references.

**Figure 3:**
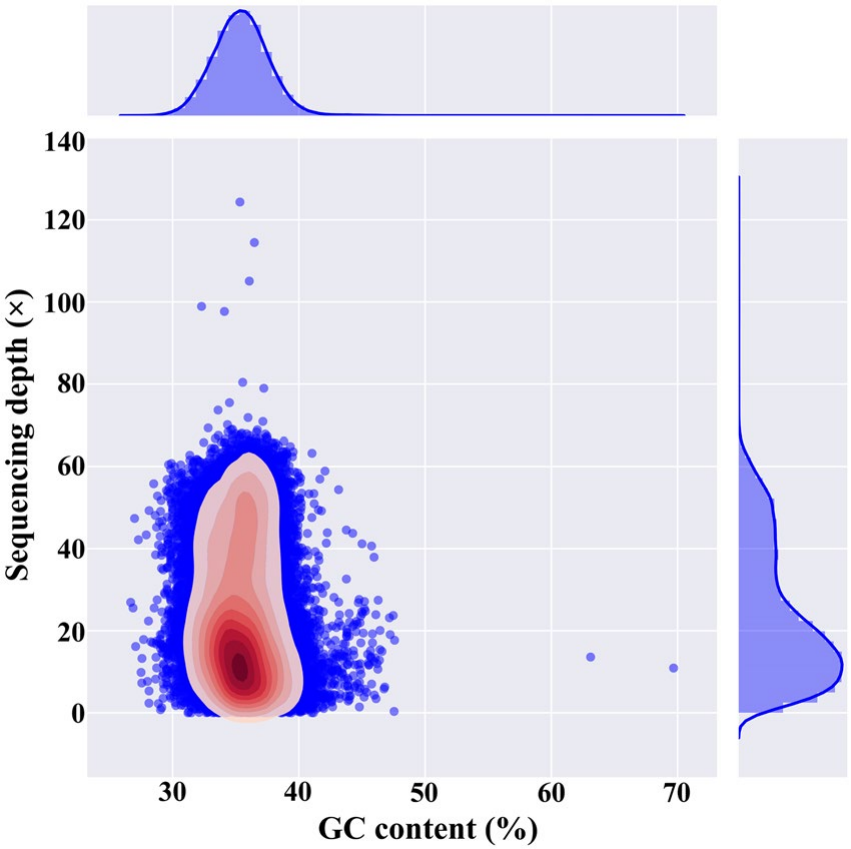
Guanine plus cytosine (GC) content and average sequencing depth of the genome data. The X-axis is the GC content percent across every 10-kb non-overlapping sliding window. The Y-axis represents the average sequencing depth. The distribution of GC content is at the top, while the distribution of sequence depth is on the right side.

Using the Illumina HiSeq Xten PE150 platform, we obtained 5,292,630,018 clean reads with a total of 778.02 Gb of data, which were used for subsequent K-mer analysis. The 17-mer frequency distribution curve exhibited two peaks at depths of 15 and 30, respectively (Fig. 2). According to the flow cytometry results, it was deducible that the second was the main peak corresponding to the expected K-mer depth. Statistical analysis showed that the total number of K-mers was 347,935,256,501. Using the formula of genome size = total K-mer number/peak depth, the genome size of this sequencing sample was estimated to be 11,597,841,883 bp. The first peak observed at 1/2 of peak depth displayed a high level of heterozygosity for this Chinese fir sample. Simulation analysis using the *Arabidopsis thaliana* genome revealed that it had a 2.0~2.1% heterozygosity rate (Fig. 2). Similarly, the content of repetitive sequences was estimated to reach 74.89%. In addition, guanine plus cytosine (GC) content analysis under different sequencing depths showed a 36.04% GC content of Chinese fir (Fig. 3).

### Assembly and identification of SSR in Chinese fir

We performed sequence assembly using clean data and obtained 10,982,272 scaffolds with an average length of 693.5 bp. There were 10,982,265 scaffolds longer than 100 bp, and 804,114 were longer than 2 kb (Table 1). The total length of the assembled sequences was 7.62 Gb, exhibiting a relatively large difference from what we estimated (11.6 Gb), suggesting that a systematic sequencing approach must be designed for the large Chinese fir genome with high heterozygosity.

**Table 1:** Statistics of the assembled scaffolds in Chinese fir.

	Size(bp)	Number
N90	273	5,649,461
N80	532	3,657,308
N70	821	2,504,998
N60	1,152	1,720,450
N50	1,566	1,151,829
Longest	62,714	—
Total Size	7,615,716,973	—
Total Number(>=100 bp)		10,982,265
Total Number(>=2 kb)		804,114

**Figure 4:**
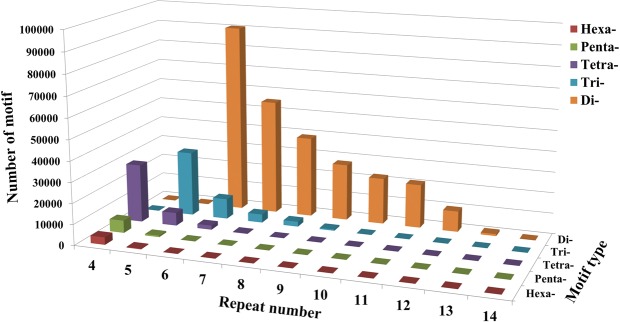
Distributions of di- to hexanucleotide motif types with different repeat numbers (from 4 to 14) in the assembled genomic sequences of Chinese fir.

**Figure 5:**
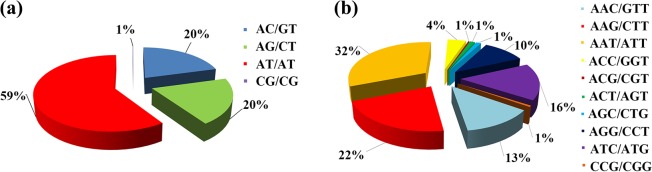
Frequency distribution of different dinucleotide and trinucleotide repeat motifs in the assembled genomic sequences of Chinese fir. (**a**) Frequency distribution of different dinucleotide repeat motifs. (**b**) Frequency distribution of different trinucleotide repeat motifs.

A total of 362,193 SSR loci were identified with the assembled sequences. These loci were distributed on 299,303 scaffolds, with one SSR locus every 13,184 bp (no counting of unknown bases). We grouped these SSR loci according to the number of nucleotides in the repeat motifs (Fig. 4). The dinucleotide motif had the largest number (266,593), accounting for 73.6%, followed by trinucleotide motifs (48,138, 13.3%). The numbers of tetra-, penta- and hexanucleotide motifs were 36,732 (10.1%), 6,791 (1.9%), and 3,939 (1.1%), respectively. The number of SSR-containing sequences decreased as the repeat number of motifs increased. In dinucleotide SSR loci, AT/TA (59.4%) was the most abundant repeat motif, and CG/CG was the least abundant (0.1%) (Fig. 5a). AAT/ATT was the most abundant trinucleotide motif (31.8%), followed by AAG/CTT (21.5%), ATC/ATG (15.9%), AAC/GTT (12.8%) and AGG/CCT (10.3%) (Fig. 5b). The tetra-, penta- and hexanucleotide SSR loci contained more motif types, each in a relatively small percentage (Supplementary Table S1).

### Development and screening for polymorphic gSSR markers

**Figure 6:**
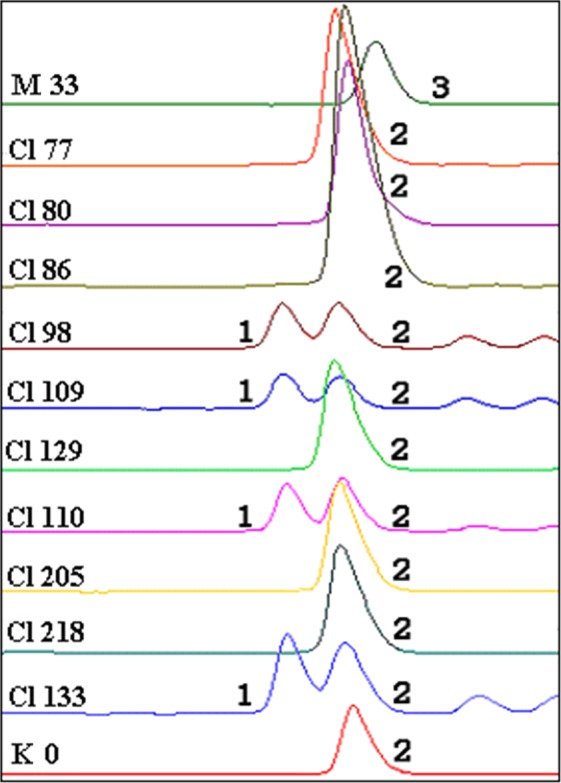
Allelic variation of gSSR marker CLgSSR1 among 12 Chinese fir clones. To reveal the allelic variation, PCR products of CLgSSR1 primer pairs were analyzed and visualized by capillary electrophoresis. The symbols, such as M33, Cl77, Cl80, etc., on the left of the picture represent different Chinese fir clones. The peaks 1, 2 and 3 represent the 160 bp, 163 bp and 166 bp, respectively.

In this study, 156 assembled sequences without any unknown base were randomly selected to design the SSR primers, and 156 pairs of primers were successfully designed. Of these primer pairs, 89 were randomly selected to test the successful rate of PCR amplification. Through analysis by 1% agarose gel electrophoresis, 79 (88.76%) SSR primer pairs generated amplification products with expected sizes. The sequences of these 79 SSR loci were deposited into NCBI (accession numbers MK948081- MK959313), and the corresponding repeat motifs and primer sequences were listed in Supplementary Table S2. In order to screen out polymorphic SSR loci, DNA samples of 12 Chinese fir clones from different geographical provenances were used as PCR templates, and capillary electrophoresis of PCR products were further performed to analyse and visualize allelic variation. As an example, three alleles were detected in these 12 clones by CLgSSR1 primers showing three peaks at 160, 163 and 166 bp, respectively (Fig. 6). In total, 46 polymorphic gSSR markers were detected by these primer pairs accounting for 58.2% of effective primers (Table 2).

**Table 2:** Novel genomic SSR loci with genetic diversity analysis in Chinese fir clones.

No.	Loci	Size range (bp)	Na	Ne	Ho	He	PIC	I*
1	CLgSSR1	139–175	5	2.690	0.065	0.630	0.547	1.093
2	CLgSSR3	138–174	6	2.524	0.169	0.605	0.533	1.076
3	CLgSSR4	129–171	7	2.287	0.388	0.564	0.558	1.158
4	CLgSSR5	131–167	4	1.881	0.000	0.469	0.353	0.704
5	CLgSSR6	128–170	7	3.301	0.352	0.699	0.647	1.360
6	CLgSSR7	138–174	4	2.562	0.000	0.611	0.550	1.034
7	CLgSSR8	128–176	6	2.938	0.065	0.661	0.604	1.240
8	CLgSSR10	169–211	12	2.683	0.333	0.629	0.593	1.427
9	CLgSSR11	139–175	10	1.991	0.191	0.499	0.464	1.138
10	CLgSSR13	137–173	7	3.057	0.468	0.674	0.610	1.344
11	CLgSSR14	130–166	7	3.353	0.250	0.703	0.658	1.412
12	CLgSSR15	140–176	6	3.031	0.218	0.672	0.616	1.250
13	CLgSSR16	141–171	4	1.606	0.051	0.378	0.342	0.657
14	CLgSSR17	145–187	17	6.658	0.880	0.852	0.833	2.150
15	CLgSSR19	138–174	6	1.382	0.088	0.277	0.251	0.573
16	CLgSSR20	124–178	6	1.834	0.313	0.456	0.438	0.955
17	CLgSSR21	127–169	6	2.802	0.348	0.645	0.604	1.238
18	CLgSSR22	133–169	7	3.756	0.430	0.736	0.684	1.489
19	CLgSSR28	172–208	2	1.726	0.370	0.422	0.358	0.612
20	CLgSSR29	126–174	4	1.275	0.139	0.216	0.223	0.467
21	CLgSSR30	137–185	9	3.214	0.439	0.691	0.654	1.509
22	CLgSSR33	142–178	3	2.297	0.259	0.566	0.464	0.923
23	CLgSSR35	130–166	4	1.411	0.083	0.292	0.304	0.538
24	CLgSSR39	132–168	5	3.043	0.000	0.673	0.637	1.268
25	CLgSSR40	143–179	6	2.206	0.148	0.548	0.492	1.024
26	CLgSSR41	136–178	8	2.400	0.493	0.585	0.566	1.203
27	CLgSSR43	128–164	5	1.683	0.074	0.407	0.403	0.755
28	CLgSSR45	132–168	3	1.944	0.000	0.487	0.391	0.766
29	CLgSSR46	127–169	6	3.175	0.140	0.687	0.624	1.309
30	CLgSSR48	119–155	7	3.068	0.149	0.676	0.629	1.331
31	CLgSSR51	180–216	7	1.603	0.098	0.377	0.305	0.766
32	CLgSSR52	110–152	8	2.623	0.412	0.620	0.563	1.206
33	CLgSSR55	144–180	3	1.245	0.028	0.197	0.189	0.412
34	CLgSSR56	149–185	6	1.974	0.005	0.495	0.445	0.928
35	CLgSSR57	124–166	6	2.228	0.461	0.553	0.533	1.163
36	CLgSSR58	149–185	13	5.624	0.678	0.824	0.802	1.903
37	CLgSSR59	134–170	5	2.106	0.370	0.526	0.504	1.048
38	CLgSSR60	145–181	8	3.068	0.347	0.676	0.604	1.268
39	CLgSSR64	136–172	3	1.896	0.116	0.474	0.384	0.735
40	CLgSSR65	139–175	7	2.598	0.257	0.617	0.531	1.128
41	CLgSSR66	155–197	4	1.196	0.056	0.165	0.179	0.358
42	CLgSSR67	116–158	7	3.122	0.389	0.681	0.632	1.343
43	CLgSSR68	145–181	15	6.440	0.421	0.847	0.823	2.122
44	CLgSSR71	135–171	6	3.136	0.278	0.683	0.638	1.327
45	CLgSSR76	130–170	7	4.923	0.750	0.850	0.770	1.750
46	CLgSSR78	135–167	4	3.460	0.125	0.758	0.658	1.305
Total			298	—	—	—	—	—
mean			6.478	2.718	0.254	0.573	0.526	1.125

Na, number of alleles; Ne, number of effective alleles; Ho, observed heterozygosity; He, expected heterozygosity; PIC, polymorphism information content; I*, Shannon’s information index.

### Diversity survey of novel gSSR loci in Chinese fir

The screened 46 polymorphic gSSR markers were subsequently used to assess their utility in studying genetic variation in 199 Chinese fir clones, and the obtained information included size range, number of alleles (Na), effective alleles (Ne), observed heterozygosity (Ho), expected heterozygosity (He), Shannon’s information index (I*) and polymorphism information content (PIC) (Table 2).

A total of 298 alleles were detected by the 46 gSSR markers in the 199 clones, and the number of alleles per locus ranged from 2 (CLgSSR28) to 17 (CLgSSR17) with an average of 6.478 alleles per locus. The Ne fluctuated between 1.20 (CLgSSR66) and 6.66 (CLgSSR17) with an average of 2.72 per locus. The locus CLgSSR68 (6.44) and CLgSSR58 (5.62) also showed relatively high Ne values, which were 6.44) and 5.62, respectively. The average of Ho was 0.25, and the Ho value of CLgSSR17 (0.88) was highest among all loci indicating highly heterozygosity of this locus, while the CLgSSR5, CLgSSR7, CLgSSR39 and CLgSSR45 (Ho = 0) were all homozygous in the detected clones. As an important indicator for measuring the genetic diversity of a population, the He of SSRs between 0.3 and 0.8 indicates that a population has higher genetic diversity^24^. The average He was 0.57, suggesting a high degree of genetic variation for these clones. The Shannon information index (I*) changed between 0.36 (CLgSSR66) and 2.15 (CLgSSR17), and its mean was 1.13. The average value of PIC was 0.53, ranging from 0.18 (CLgSSR66) to 0.83 (CLgSSR17). Based on the classification of Botstein *et al*.^25^, 29 loci (63.04%) with PIC values > 0.5 were highly informative, 12 (26.09%) loci were reasonably informative (0.25 < PIC < 0.5), and the others were slightly informative (0 < PIC < 0.25). These results indicated that there was a relatively high level of genetic diversity in this breeding population composed of Chinese fir clones.

### Genetic diversity analysis of Chinese fir clones

**Figure 7:**
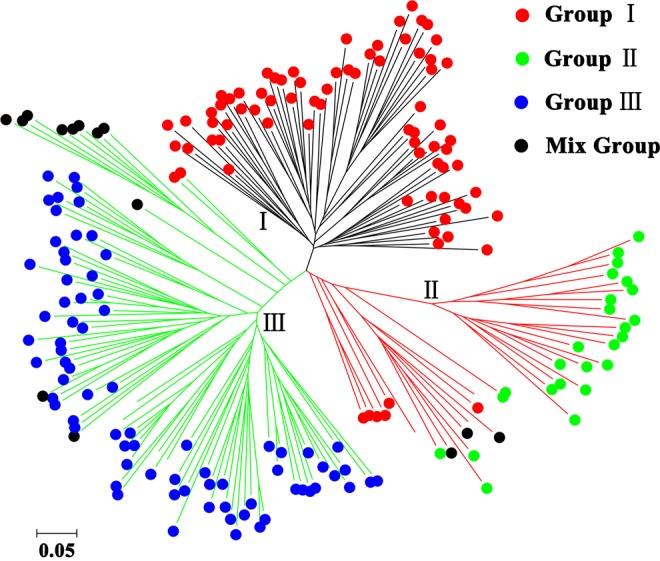
Neighbor-joining dendrogram of 199 Chinese fir clones based on Nei’s 1983 genetic distances. To compare with population structure revealed by STRUCTURE, those clones belonging to the different groups inferred by STRUCTURE analysis were indicated by different colored dots, respectively.

**Figure 8:**
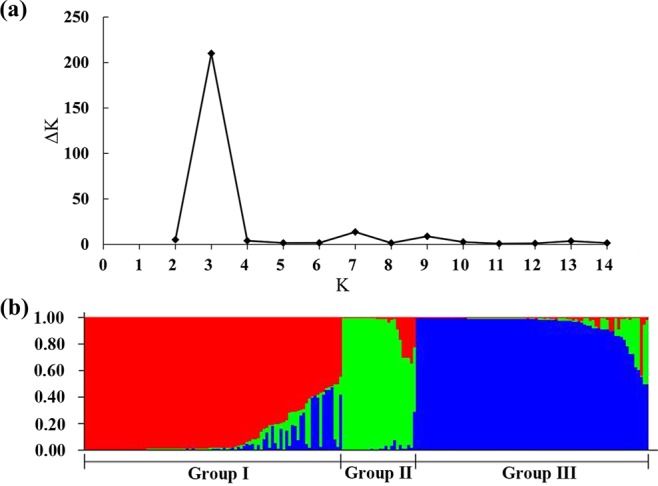
Population structure analysis of 199 Chinese fir clones using STRUCTURE software. (**a**), ΔK was calculated to determine the most appropriate K value for population structure estimation. The modal value of this distribution is the true K (*) or the uppermost level of structure, here three (K = 3) clusters. (**b**) Population structure of 199 Chinese fir clones. These clones were assigned into three groups (Groups I, II and III) as indicated by the different coloured blocks. Each clone was represented by a single colored line. The greater proportion of a color, the greater the possibility that the represented individual belongs to the group indicated by that color.

As mentioned above, all these polymorphic SSR loci were used to estimate the genetic diversity of 199 Chinese fir clones. At first, genetic distance was calculated through pairwise comparisons of the 199 clones by PowerMarker to analyse the genetic similarity between different clones. As a result, the Nei’s genetic distances ranged from 0.0701 to 0.8609 with an average of 0.5073. And most pairwise genetic distances (66.01%) were between 0.4 and 0.6, which indicated high genetic variation among these Chinese fir clones. Moreover, the neighbor-joining (NJ) dendrogram based on Nei’s genetic distances revealed that all clones analysed could be grouped into three major clusters I, II, and III comprised of 70, 37, and 92 clones, respectively (Fig. 7, Supplementary Table S3). On the other hand, to reveal the genetic composition of each clone, we also performed structure analysis based on these 46 polymorphic gSSR loci. According to the method of Evanno *et al*.^26^, ΔK was introduced to determine the most appropriate K value, which represents the most likely number of groups. As shown in Fig. 8a, the ΔK value was the highest when K was set to 3. It suggested that these Chinese fir clones were also classified into 3 groups (Fig. 8b). As expected, that most clones were assigned into the same group by clustering of these two methods (Fig. 7, Supplementary Table S3).

According to the report of Wu *et al*.^27^, we further analysed the membership probabilities (Q) of Chinese fir clones in the different groups. A total of 184 (92.5%) clones with Q ≥ 0.6 were classified into three groups and were regarded as having a relatively simple genetic structure. Group I had the largest number of clones (80, 40.2%), and it’s worth noting that most of the clones (29/30) from Hunan province were classified into this group. Twenty-five (12.6%) clones belonging to the Groups II were all from Guangxi province. The third group (Group III) contained 79 (39.7%) clones from Rongshui (46), Sanjiang (18), and three other counties (15) of Guizhou province (Supplementary Table S3). The other clones with complicated and mixed origins were placed into the mixed group (Supplementary Table S3). Clones of the same geographical provenance exhibited different genetic compositions. For example, of all clones from Rongshui, Guangxi, 84 were classified into 3 different groups, while the remaining 3 clones belonged to the mixed group. Fifteen clones from Napo, Guangxi were distributed in Groups I and II. Furthermore, some clones from different geographical provenances were classified in the same group suggesting that their genetic background might be similar. Interestingly the mixed group was mainly consisted of 8 clones from Zhejiang and Fujian provinces, which all showed admixture with Groups I and III (Fig. 7). According to the model-based clustering results, an analysis of molecular variance (AMOVA) was conducted and the genetic differentiation coefficient (*F*st) was calculated to investigate population differentiation. The results showed that 21.33% (*P* < 0.001) of the total molecular variation was partitioned among groups, while 38.68% (*P* < 0.001) of the variation resulted from genetic differences among subgroups and 39.99% (*P* < 0.001) within subgroups. The pairwise *F*st values of the three inferred groups were 0.23 (Group I and II, *P* < 0.001), 0.19 (Group I and III, *P* < 0.001) and 0.25 (Group II and III, *P* < 0.001), respectively. This suggested that these three groups were statistically distinguished from each other. In addition, the gene flow (*Nm*) was estimated to be 0.92 based on the method of Slatkin *et al*.^28^, indicating a limited gene flow among these inferred groups.

## Discussion

### Genome characteristics of Chinese fir

Based on genome survey sequencing data, we could estimate the genome size of a non-model plant species using K-mer analysis. In the past few years, this approach has been successfully applied to the genomic analysis of some woody plants, such as Chinese bayberry (*Myrica rubra*)^29^, Chinese jujube (*Ziziphus jujuba*)^30^, and pistachio^20^. In this study, for the first time, we conducted genome survey sequencing on Chinese fir and obtained 778.02 Gb of clean data. The 17 K-mer and flow cytometry analyses showed that the Chinese fir genome was approximately 11.6–13.2 Gb, which is smaller than the Norway spruce (*Picea abies*) genome (19.6 Gb)^31^ and larger than the ginkgo (*Ginkgo biloba*) genome (10.0 Gb)^32^. The K-mer analysis also revealed a high level of heterozygosity for the sequencing sample, which was probably due to a high natural outcrossing rate in Chinese fir. The GC content can affect the quality of Illumina sequencing and subsequent assembly^33^. The GC content of this Chinese fir sample was close to that of wild sweet potato (36%)^34^ and significantly lower than that of Chinese jujube (48%)^30^. In addition, the repeat rate was slightly higher than that of elephant grass (71.36%)^21^. Such information on the Chinese fir genome may provide an important reference for whole-genome sequencing and the selection of assembly strategies in subsequent steps.

### Characteristics of gSSR loci in Chinese fir

A total of 362,193 SSR loci were detected from the Chinese fir genome survey sequencing data. The average occurrence frequency of SSR loci was 13.18 kb, which was lower than that in rice (2.75 kb), *A. thaliana* (2.39 kb), sesame (11.69 kb)^18^ and pistachio (8.67 kb)^20^. Among SSR motifs, dinucleotide repeats were the most frequent (73.6%), with AT/AT accounting for 59.4%. Next were the trinucleotide repeats (13.3%), with AAT/ATT accounting for 31.8%. This was similar to the SSR distribution characteristics in the genomes of sesame^18^, buckwheat^19^, and pistachio^20^. However, among the EST-SSRs in Chinese fir, the trinucleotide repeats were the most abundant^12,13^, suggesting that most of the dinucleotide repeats may be from the intron regions. The massive sequences from the genome survey sequencing provided sufficient data for the identification of high-quality polymorphic SSR loci. The polymorphic SSR markers obtained in this study accounted for 58.2% of the detectable markers, significantly higher than those in Chinese bayberry (31.0%)^29^ and *Metasequoia glyptostroboides* (32.9%)^35^. Of these polymorphic markers, 29 were highly polymorphic (PIC > 0.5).

### Genetic diversity and population structure of Chinese fir clones

Polymorphic gSSR markers were used to evaluate the genetic diversity of 199 Chinese fir clones. The average Na, I* and PIC were calculated to be 6.48, 1.125 and 0.526, respectively. Compared to findings from Ouyang and Qi *et al*.^14,36^, the breeding resources of Chinese fir used in this study had a higher level of genetic diversity. The model-based population structure analysis could classify the 199 clones into 3 groups, consistent with the results from an NJ method based on Nei’s genetic distance. And, both AMOVA and *F*st analyses confirmed the population structure and its statistical significance. The results of the population structure analysis also revealed the existence of introgression and gene flow among different clones. Three groups were identified by two methods, which did not match the clones’ geographical origins. This was in agreement with previous reports regarding Chinese fir^37,38^, which could be related to wind pollination and a high natural outcrossing frequency in this species, as well as to artificial germplasm exchange. Therefore, the selection of Chinese fir breeding resources should not only focus on geographical origin but also consider the genetic structure of the population.

To gain knowledge on the genome structure of Chinese fir, we had more than 30× Illumina data coverage for its genome survey. The assembled sequences were also used to search for SSR loci, to develop novel gSSR markers, and to study genetic diversity and population structure in this important species. Flow cytometry and K-mer analysis indicated that the Chinese fir genome is 11.6–13.2 Gb in size. The sequencing sample was highly heterozygous and had a high level of repeats, and its GC content was approximately 36.04%. Overall, 362,193 SSR loci were found with a frequency of 13.18 kb. Forty-six polymorphic gSSR primer pairs were developed, and 298 alleles were successfully amplified from 199 Chinese fir clones. These Chinese fir resources could be divided into three groups. In conclusion, in this study, we present the data on the genome structure of the Chinese fir, which may help in designing further whole genome sequencing strategies. Furthermore, we also developed novel polymorphic gSSR markers, which will facilitate germplasm characterization, genetic diversity and population structure studies in this important species.

## Materials and Methods

### Plant materials and DNA extraction

Chinese fir clone “ZL06” is one of the superior clones with faster growth rate and stronger resistance, and has been widely cultivated as commercial forest in Zhejiang province of China. Due to its commercial value and desirable traits, ZL06 was chosen as material for genome sequencing. Three-year-old trees of this clone was grown in the Pingshan experimental station, Zhejiang A&F University, Hangzhou City, China (30 °15′36.6″N, 119 °42′13.1″E) for genome survey sequencing and flow cytometry analysis. And 199 other Chinese fir clones grown in the Hengzhi germplasm repository (29 °7′24.6″N, 118 °25′57.1″E), Kaihua County, Zhejiang Province, China, were used to test SSR markers for polymorphism and to assess genetic diversity. The numbering and geographical origins of these clones are listed in Supplementary Table S3. The tender leaves, used for DNA isolation, were collected between 21 and 27 of June 2017. After cleaning and disinfection with 70% alcohol, leaves were stored in liquid nitrogen. In addition, the *P. trichocarpa* and barley tissue culture seedlings used for the flow cytometry analysis were a generous gift from the Zhejiang University Institute of Genetics.

The genomic DNA was extracted from the leaf samples using the CTAB (Cetyltrimethyl- ammonium bromide) method^39^. DNA purity and concentration were assessed using 1% agarose gel electrophoresis and a NanoDrop 2000 spectrophotometer (Thermo Fisher Scientific, Inc., Wilmington, DE, USA). DNA samples were diluted to a concentration of 50 ng/μl for the following SSR-polymerase chain reaction (PCR).

### Flow cytometry analysis

The genome size was determined using the CytoFLEX Flow Cytometer (Beckman Coulter, Inc., Brea, CA, USA). A total of 60 mg of fresh leaf tissue was harvested and placed on a Petri dish. One millilitre of lysis buffer (MgSO_4_ buffer) was added to the Petri dish, and the leaf tissue was cut into pieces with a sharp blade. The mixture was filtered and collected in a 1.5 ml centrifuge tube and centrifuged for 5 min at 1000 rpm/min. The supernatant was discarded, and the pellet was resuspended in 100 μl of pre-chilled lysis buffer. An aliquot of 150 μl of pre-chilled PI staining solution (50 μg/ml) was added for the fluorescence labelling of nuclear DNA. The sample was incubated in the dark at 4 °C for 5 min and then filtered and loaded onto the flow cytometer for detection. The detection for each sample was performed with ten replicates. CyExpert software (Beckman Coulter, Inc.) was used for capturing fluorescent signals and data analysis. Barley (*H. vulgare* ‘Golden promise’, 2 C = 10.43 pg) and *P. trichocarpa* (Nisqually-1, 2 C = 0.98 pg) with a known genome size served as reference standards. The relative nuclear DNA content of Chinese fir (*C. lanceolate* ‘ZL06’) was measured according to o the following formula: Sample 2C-value (pg) = (Sample peak mean/Standard peak mean) * nuclear DNA content of the reference Standard (pg). Genome size was estimated based on the conversion formula: 1 pg DNA = 0.978 × 10^9^ bp^40^.

### Illumina sequencing and K-mer analysis

The genomic DNA of clone ZL06 was randomly fragmented into 350 bp inserts, and a DNA sequencing library was constructed. Then, sequencing (paired-end) was performed at Nextomics Bioscience Co., Ltd. (Wuhan, China). After removing low-quality reads, clean reads were obtained and used for K-mer analysis. The sequence data were deposited in the Genome Sequence Archive^41^ in BIG Data Center^42^, Beijing Institute of Genomics (BIG), Chinese Academy of Sciences, under accession numbers CRA001775. We used JELLYFISH 2.1.4 to conduct the K-mer analysis and obtained the corresponding frequency distributions^43^. Based on k-mer frequency distributions, genome size was calculated using the information on peak depth and the number of 17-mer. The heterozygosity rate was also estimated according to k-mer number at half the peak depth and simulation analysis with *A. thaliana* genome data. The proportion of repeated sequences was analysed using GENOMESCOPE software^44^.

### Sequence assembly and SSR identification

Genome assembly using sequencing data was performed with the SOAPdenovo v2.01 software with the default settings^45^. For the assembly, contigs were firstly obtained according to the De-Brujin-Graph (DBG) algorithm. After mapping the paired-end reads back to resultant contigs, scaffolds were constructed step by step. Finally, the gaps inside the scaffolds were filled using paired-end reads. SOAPaligner v2.21 was used to analyse the GC-depth distribution^46^. The scaffolds longer than 100 bp in length were retained. Then, the GC average sequencing depth was calculated by the 10-kb non-overlapping sliding windows along the assembled sequences.

SSR loci were searched using the Perl script MISA (http://pgrc.ipk-gatersleben.de/misa/misa.html). The search parameters were set for the identification of di-, tri-, tetra-, penta-, and hexanucleotide motifs with a minimum number of 6, 5, 4, 4, and 4 repeats, respectively. The numbers of SSR repeats, frequencies of SSRs and motif types were collected and statistically analyzed using Excel 2016 (Microsoft, WA, USA). Web-based BatchPrimer3 v1.0 (http://probes.pw.usda.gov/batchprimer3/) was used for designing SSR primers. The detailed parameters were as follows: 100–300 bp final product length (optimal 150 bp), primer size from 18 to 23 bp (optimal 21 bp) and GC content 40–70% (optimal 50%); the primer melting temperature was set between 50 °C and 70 °C (optimal 55 °C). All primers were synthesized by GenScript Co., Ltd. (Nanjing, China).

### PCR and capillary electrophoresis

SSR-PCR was performed in a total volume of 10 μl containing 50 ng DNA, 1× SuperMix containing Taq DNA polymerase (Takara, Dalian, China), and 2 pmol of each primer. The PCR program was as follows: initial denaturation at 94 °C for 5 min, followed by 32 cycles at 94 °C for 30 s, 48~54 °C (determined by primer Tm) for 30 s and 72 °C for 40 s and a final extension at 72 °C for 10 min. Amplified products were first checked using 1% agarose gel electrophoresis. Products with expected sizes were then subjected to capillary electrophoresis. All detections were made in three replicates. SSR alleles were visualized and scored with the Q-Analyzer-1.3.5.1 software (BiOptic Inc.).

### Data analysis of genetic diversity and population structure

The 46 polymorphic primer pairs were used to evaluate the genetic diversity of the Chinese fir clones. Parameters including Na, Ne, Ho, He, and I* were analysed with the POPGENE software version 1.32^47^. The PIC value of each SSR locus was calculated based on the formula^25^.

$$PIC=1-\mathop{\sum }\limits_{i=1}^{n}\,{P}_{i}^{2}-\mathop{\sum }\limits_{i=1}^{n-1}\,\mathop{\sum }\limits_{j=i+1}^{n}\,2{P}_{i}^{2}{P}_{j}^{2}$$

where *Pi* and *Pj* are the frequency of the *i*th and *j*th allele for the given locus, respectively.

Two different approaches were used to assess population structure of Chinese fir clones. The first one was Bayessian clustering analysis implemented with STRUCTURE 2.3.4 software to determine the most likely number of group and assign clones to their most likely group^48^. The number of groups was set as K (K = 1~15), and each run was replicated 20 times to ensure consistency of results. The burn-in period and Markov chain Monte Carlo (MCMC) were set at 50,000 and 500,000, respectively. The true number of group was determined by the logarithm of likelihood for each K, L(K), and the optimum value ΔK was obtained by the formula^26^.

$$\Delta K=\frac{{\rm{mean}}\,(|{\rm{L}}(K\,+\,1)-2\,{\rm{L}}({\rm{K}})+{\rm{L}}({\rm{K}}-1)|)}{{\rm{s}}[{\rm{L}}({\rm{K}})]}$$

where s[L(K)] is the standard deviation of L(K). The Q data of the 20 replicate runs of the best value of K were integrated by CLUMPP software^49^. The second clustering was based on Nei’s 1983 genetic distance and on the NJ algorithm using PowerMarker v3.25 software^50^. This bootstrap analysis was carried out with 1,000 replicates. And MEGA 7.0 software was used to plot the dendrogram^51^. In addition, to summarize the major patterns of variation within the multi-locus dataset, an AMOVA analysis was performed using GenAlEx V6.5 with 999 permutations^52^. *Nm* of whole population was also estimated using the formula^28^.

$$Nm=\frac{1}{4}\left(\frac{1}{Fst}-1\right)$$

## Supplementary information

Supplementary information

## Data Availability

The sequence data reported in this study have been deposited in the Genome Sequence Archive in BIG Data Center, Beijing Institute of Genomics (BIG), Chinese Academy of Sciences, under accession numbers CRA001775, CRA001775 that are publicly accessible at http://bigd.big.ac.cn/gsa.
