# Supplementary material for: Genome survey of Chinese fir (Cunninghamia lanceolata): Identification of genomic SSRs and demonstration of their utility in genetic diversity analysis
Source: Sci Rep. 2020 Mar 13;10:4698. doi: 10.1038/s41598-020-61611-0 (PMC7070021; doi:10.1038/s41598-020-61611-0)
Supplement: Supplementary file 1 — Supplementary information [file 41598_2020_61611_MOESM1_ESM.pdf]

Genome survey of Chinese fir (*Cunninghamia lanceolata*): Identification of genomic SSRs and demonstration of their utility in genetic diversity analysis

Erpei Lin[1, 3], Hebi Zhuang[1, 3], Jingjian Yu[1], Xueyu Liu[1], Huahong Huang [1]*, Muyuan Zhu[2], Zaikang Tong[1]*

[1] The State Key Laboratory of Subtropical Silviculture, Institute of Biotechnology, College of Forestry and Biotechnology, Zhejiang A & F University, Lin'an, Hangzhou 311300, Zhejiang, China

[2] Key Laboratory for Cell and Gene Engineering of Zhejiang Province, Institute of Genetics, College of Life Sciences, Zhejiang University, Hangzhou 310058, Zhejiang, China

[3] These authors contributed equally to this work

Email: Erpei Lin - zjulep@hotmail.com

Huahong Huang - huanghh@zafu.edu.cn

Zaikang Tong - zktong@zafu.edu.cn

* Corresponding author

Table S1 Distribution of different tetra-, penta- and hexanucleotide repeat motifs in Chinese fir genome.

Tetranucleotide repeat motifs		Pentanucleotide repeat motifs						Hexanucleotide repeat motifs									
Motif type	Count	Motif type	Count	Motif type	Count	Motif type	Count	Motif type	Count	Motif type	Count	Motif type	Count	Motif type	Count	Motif type	Count
AAAT/ATTT	2692	AAAAT/ATTTT	226	AAGTG/ACTTC	3	AGGAT/ATCCT	1	AAAAAG/CTTTTT	9	AAACCC/GGGTTT	3	ACAGGG/CCCTGT	2	AAGGAC/CCTTGT	1	ACTCCT/AGGAGT	1
ACAT/ATGT	2570	AAAAC/GTTTT	86	ACCAT/ATGGT	3	AGGCC/CCTGG	1	AAAAAT/ATTTTT	9	AAATCC/ATTTGG	3	ACATAG/ATGTCT	2	AAGGCG/CCTTCG	1	ACTCGG/AGTCCG	1
AGAT/ATCT	571	AATAT/ATATT	80	ACGGG/CCCGT	3	AGGCG/CCTCG	1	AGAGGC/CCTCTG	8	AACGGC/CCGTTG	3	ACATCT/AGATGT	2	AAGGGC/CCCTTG	1	ACTGAG/AGTCTC	1
AAAG/CTTT	504	AAGAG/CTCTT	30	CCCCG/CGGGG	3	CCGCG/CGCGG	1	AGAGGG/CCCTCT	8	AACTAT/AGTTAT	3	ACCAGT/ACTGGT	2	AAGGGT/ACCCTT	1	ACTGCC/AGTGGC	1
AATG/ATTC	432	AATAG/ATTCT	30	AACTC/AGTTG	2	Total	771	ACACCC/GGGTGT	7	AAGGAG/CCTTCT	3	ACCCCT/AGGGGT	2	AAGGTC/ACCTTG	1	ACTGCT/AGCAGT	1
AATT/AATT	427	AAATT/AATTT	27	AACTG/AGTTC	2			AACACC/GGTGTT	6	AAGTGT/ACACTT	3	ACCTGC/AGGTGC	2	AAGGTG/ACCTTC	1	AGAGAT/ATCTCT	1
AATC/ATTG	384	AAAAG/CTTTT	26	AATAC/ATTGT	2			ACCCTC/AGGGTG	6	AATATG/ATATTC	3	ACGATG/ATCGTC	2	AAGTGG/ACTTCC	1	AGATCC/ATCTGG	1
AAAC/GTTT	358	AAATC/ATTTG	23	ACACT/AGTGT	2			ACTCCC/AGTGGG	6	ACACAT/ATGTGT	3	ACGGGG/CCCCGT	2	AATAGT/ACTATT	1	AGGCAT/ATGCCT	1
AGGG/CCCT	147	AGGGG/CCCCT	15	ACAGG/CCTGT	2			AGATGG/ATCTCC	6	ACACTC/AGTGTG	3	ACTAGC/AGTGCT	2	AATATC/ATATTG	1	AGGCGG/CCGCCT	1
ATCC/ATGG	91	AAACT/AGTTT	13	ACATC/ATGTG	2			AGGGGC/CCCCTG	6	ACATCC/ATGTGG	3	ACTATG/AGTCAT	2	AATATT/AATATT	1	AGGGAT/ATCCCT	1
AAGG/CCTT	63	AAAGG/CCTTT	13	ACCCT/AGGGT	2			AAAAAC/GTTTTT	5	ACCCGC/CGGGTG	3	ACTCTC/AGAGTG	2	AATCCT/AGGATT	1	AGGGCG/CCCTCG	1
AACT/AGTT	56	AATCC/ATTGG	13	ACGAG/CGTCT	2			AAATAT/ATATTT	5	ACCTCG/AGGTCG	3	AGCCCG/CGGGCT	2	AATCTC/AGATTG	1	ATGCCC/ATGGGC	1
ACTC/AGTG	56	ATCCC/ATGGG	13	ACGCC/CGTGG	2			AAATGG/ATTTCC	5	ACCTCT/AGAGGT	3	AGCGGG/CCCGCT	2	AATGAG/ATTCTC	1	Total	341
ACGC/CGTG	37	ACTCT/AGAGT	11	AGCCG/CGGCT	2			AACATC/ATGTTG	5	AGCAGG/CCTGCT	3	AGGGGG/CCCCCT	2	AATGGC/ATTGCC	1		
ACCT/AGGT	35	AGAGG/CCTCT	11	ATCGC/ATGCG	2			AACTCT/AGAGTT	5	AAACAC/GTGTTT	2	AAAACC/GGTTTT	1	AATGGG/ATTCCC	1		
AACC/GGTT	34	AAATG/ATTTC	10	ATGCC/ATGGC	2			AAGTAG/ACTTCT	5	AAACAG/CTGTTT	2	AAAAGT/ACTTTT	1	AATGTG/ACATTC	1		
ACAG/CTGT	29	ACCCC/GGGGT	9	AAAGC/CTTTG	1			ACACGC/CGTGTG	5	AAAGAG/CTCTTT	2	AAAATG/ATTTTC	1	AATTAC/AATTGT	1		
AGGC/CCTG	13	AAAGT/ACTTT	8	AACCT/AGGTT	1			ACATAT/ATATGT	5	AAATTC/AATTTG	2	AAACTC/AGTTTG	1	ACAGTC/ACTGTG	1		
ATGC/ATGC	11	AAACC/GGTTT	7	AACGT/ACGTT	1			AGATAT/ATATCT	5	AACAAT/ATTGTT	2	AAAGAC/CTTTGT	1	ACCACG/CGTGGT	1		
AAGC/CTTG	9	AACCC/GGGTT	7	AAGAC/CTTGT	1			AAAGGG/CCCTTT	4	AACACG/CGTGTT	2	AAAGTG/ACTTTC	1	ACCAGC/CTGGTG	1		
ACCC/GGGT	8	AATCT/AGATT	6	AAGAT/ATCTT	1			AACCTC/AGGTTG	4	AACCCG/CGGGTT	2	AAATAC/ATTTGT	1	ACCCAG/CTGGGT	1		
ACGT/ACGT	7	AATTC/AATTG	6	AAGCC/CTTGG	1			AACTCC/AGTTGG	4	AACCCT/AGGGTT	2	AAATAG/ATTTCT	1	ACCCAT/ATGGGT	1		
ACTG/AGTC	7	AGGGC/CCCTG	6	AAGCG/CGCTT	1			AAGAGG/CCTCTT	4	AACTTG/AAGTTC	2	AACAAG/CTTGTT	1	ACCCCC/GGGGGT	1		
CCCG/CGGG	5	AGATG/ATCTC	5	AATGT/ACATT	1			AAGGGG/CCCCTT	4	AAGAAT/ATTCTT	2	AACAGC/CTGTTG	1	ACCCTG/AGGGTC	1		
AGCC/CTGG	4	AACAC/GTGTT	4	ACACC/GGTGT	1			AATTAG/AATTCT	4	AAGATG/ATCTTC	2	AACCAG/CTGGTT	1	ACCGAT/ATCGGT	1		
ACCG/CGGT	3	AACAT/ATGTT	4	ACCAG/CTGGT	1			ACCATC/ATGGTG	4	AATACC/ATTGGT	2	AACCGG/CCGGTT	1	ACCGCC/CGGTGG	1		
AACG/CGTT	2	AAGTC/ACTTG	4	ACCTC/AGGTG	1			ACCTCC/AGGTGG	4	AATCAC/ATTGTG	2	AACTAC/AGTTGT	1	ACCGTC/ACGGTG	1		
AAGT/ACTT	2	AATGG/ATTCC	4	ACCTG/AGGTC	1			AGCCTC/AGGCTG	4	AATCAT/ATGATT	2	AAGACC/CTTGGT	1	ACGACT/AGTCGT	1		
AGCG/CGCT	2	ACTAT/AGTAT	4	ACTGC/AGTGC	1			AGGATG/ATCCTC	4	AATGAT/ATCATT	2	AAGACG/CGTCTT	1	ACGAGG/CCTCGT	1		
ATCG/ATCG	2	ATATC/ATATG	4	AGAGC/CTCTG	1			ATCCCC/ATGGGG	4	AATTAT/AATTAT	2	AAGAGC/CTCTTG	1	ACGCGC/CGCGTG	1		
ACGG/CCGT	1	AACAG/CTGTT	3	AGATC/ATCTG	1			AAAATC/ATTTTG	3	AATTCC/AATTGG	2	AAGCAG/CTGCTT	1	ACGCTC/AGCGTG	1		
AGCT/AGCT	1	AAGGC/CCTTG	3	AGCGG/CCGCT	1			AAAATT/AATTTT	3	ACAGAG/CTCTGT	2	AAGCCC/CTTGGG	1	ACGGAG/CCGTCT	1		
Total	8563	AAGGG/CCCTT	3	AGCTC/AGCTG	1			AAACAT/ATGTTT	3	ACAGCC/CTGTGG	2	AAGCCT/AGGCTT	1	ACTATC/AGTGAT	1		

Table S2 Primer sequences, repeat motifs, repeat types and product sizes of 79 novel gSSR loci developed from Chinese fir clone "ZL06".

No.	SSR locus	GenBank accession number	Sequence (5′–3′)	Sequence (5′–3′)	Repeat Motif	Repeat Type	Product size (bp)
1	CLgSSR1	MK948081	F:GACTGAATCTCAATGGTGTTG	R:CAATTCTTTCCTCCTGCATA	(TTG)6	Perfect	157
2	CLgSSR3	MK959236	F:AGGACTCCCTAGCCTTAGATT	R:AGTGGGAGGTAGAACTTGTGT	(AAG)6	Perfect	156
3	CLgSSR4	MK959237	F:GACACAAGCAAACATCCTTT	R:TGTAAGGCTGACTTCATAGAGA	(TTA)7	Perfect	150
4	CLgSSR5	MK959238	F:CCTAGTGATAGGTGGGAAGTT	R:TAAGCATAGTCACCCAATAGC	(TGG)6	Perfect	149
5	CLgSSR6	MK959239	F:CCACATCTACTTGTTGGTTGT	R:AAGGAGAAGGAAGTAGTGGAA	(TCT)7	Perfect	149
6	CLgSSR7	MK959240	F:AAACAATCCACCTCTGTCTG	R:GGAGTTTCCTTTAAGTCCTGA	(CCA)6	Perfect	156
7	CLgSSR8	MK959241	F:AGCATGTCTAGAGAATGGACA	R:TGAGAAACCACTCTACATTGC	(TAT)8	Perfect	152
8	CLgSSR9	MK959242	F:AGTCTGAGCGTACATTTCTCA	R:TATGTAAGTAATCCGCTACGC	(AGGCAG)3	Perfect	146
9	CLgSSR10	MK959243	F:CTTTGAGCTCTCCTAGTCTCC	R:ATGTACCACTTAGCGACTCTG	(CAT)7	Perfect	190
10	CLgSSR11	MK959244	F:CTTACACGCCTTCTATCTTCA	R:ACACCTTGTGCTTATCTGTGT	(GCT)6	Perfect	157
11	CLgSSR12	MK959245	F:CCATTCAGCAAAGAGTTGTAG	R:GTGCACAATAAGACAGTGGAG	(TGC)6	Perfect	149
12	CLgSSR13	MK959246	F:TAAACCACCAACTCACCATAG	R:GCAAAGCTCCATTTGATAAG	(TTG)6	Perfect	155
13	CLgSSR14	MK959247	F:CGTTAAGTCTCTAAGGCATTG	R:ATATCAGTCTGGTCAGGGTTC	(CAT)6	Perfect	148
14	CLgSSR15	MK959248	F:GCCTTATCCTGATTCAAGTCT	R:AGATTCAATGGTGTACTGGTG	(AGA)6	Perfect	158
15	CLgSSR16	MK959249	F:GTACCTTGGAATTTGTCAGC	R:TGTTAACCAATCGGTCTGTAG	(ATT)5	Perfect	156
16	CLgSSR17	MK959250	F:CAGCCATCACAGTGAGTTAAT	R:TCAGACTGGTGAGACTTGTTT	(TTA)7	Perfect	166
17	CLgSSR18	MK959251	F:ATAGCGACAGTAATCTCAGCA	R:CCTCTGTAATTCCACAGAGAA	(TAA)6	Perfect	139
18	CLgSSR19	MK959252	F:ACAGGTTGAGAGTTTCTTGGT	R:GAGTATAAGCCAAGCCAACA	(TGC)6	Perfect	156
19	CLgSSR20	MK959253	F:AACCACACTAGTAGCCTAGCA	R:GATGAAGGGTCACAAGATACA	(GAG)9	Perfect	151
20	CLgSSR21	MK959254	F:CTAAGAGGGATACAGGGAAAC	R:GGCTAGTGTAACTTGTGTTGC	(AAG)7	Perfect	148
21	CLgSSR22	MK959255	F:CAATAGCTGGCTTCTAAACAG	R:GCATCATAGATCAGGAGTTTG	(GCA)6	Perfect	151
22	CLgSSR23	MK959256	F:CATCCACGTAGATCTCAATTC	R:ACAGCAGAACAGAACAGAAGA	(GAT)6	Perfect	146
23	CLgSSR24	MK959257	F:CCTTTAGGAGATGAAGGTTCT	R:TAAGGATAAGCCCAAGAAGAT	(TACTTC)3	Perfect	126
24	CLgSSR25	MK959258	F:CTCTGTCGTTCATGTAGCTTT	R:GTGATGAATGGTATGATGCTT	(GTAGCA)3	Perfect	148
25	CLgSSR27	MK959259	F:AAATGAACAAGAGGAGCAGTA	R:GGTCTTTCATGTGTTTGTCAG	(TGAAA)3	Perfect	151
26	CLgSSR28	MK959260	F:TTCCAGGAGTATTTCTTCCTC	R:ATCATCATCATCATCATCTGC	(CAGCAA)3	Perfect	190
27	CLgSSR29	MK959261	F:ATGCTAATGGTGATAGTGGTG	R:GCTTCTCCGTTAATCTCTCC	(GAAGAG)4	Perfect	150
28	CLgSSR30	MK959262	F:AACCGACACCATCTAAGAAAC	R:GAACATGCTTCAAAGTCCATA	(CCG)8	Perfect	161
29	CLgSSR32	MK959263	F:TAAACGAATATGGATGACCTC	R:CCTTCTATTCCTTCTTCATTCA	(TTA)7	Perfect	147
30	CLgSSR33	MK959264	F:GTTGCCAAAGTTCAGAAACTA	R:TTTCTTGTCCATTAGATCGTG	(AAT)6	Perfect	160
31	CLgSSR34	MK959265	F:TCAAAGCTTTAGTCTGTGACC	R:TCTCAATTTAGTGGGAGTTGA	(CAT)6	Perfect	151
32	CLgSSR35	MK959266	F:ATGTGACTCCCATGTTGTTAG	R:TTGAGGTACATTAGGTTGCAT	(TCA)6	Perfect	148
33	CLgSSR36	MK959267	F:TAACCCTTTGTCTGATGATGA	R:CTCTGAGGATTTATGCTGCTA	(GGA)6	Perfect	147

continued the table

34	CLgSSR37	MK959268	F:TTCCACCAAATTCACTACAAG	R:TACAAGGCTGAAAGACATGAT	(GAA)6	Perfect	142
35	CLgSSR38	MK959269	F:TTCATATCATCCCATCTTCAC	R:CATGTCGTTCTGTTCTTTGAT	(TTA)6	Perfect	169
36	CLgSSR39	MK959270	F:CACCTGATTTGAACATACTCC	R:ATTTGCAGATCATGACAGAGT	(TGA)6	Perfect	150
37	CLgSSR40	MK959271	F:GTATCGTTTCTCCGTCAGATT	R:CGATGGGATAGCTACAGAAC	(GAA)6	Perfect	161
38	CLgSSR41	MK959272	F:TCCAAGAACTGTCAAATCACT	R:TAAAGTCCCAGTGAGATGTGT	(CTG)7	Perfect	157
39	CLgSSR43	MK959273	F:TTAGGCCCTCTCTACTCTTGT	R:AGACGTAGGACCATAGGTAGG	(TCA)6	Perfect	146
40	CLgSSR45	MK959274	F:GCCGAGGATAAACTATTAAGG	R:TTGTCCTCTTCATCCTAGCTT	(TGA)6	Perfect	150
41	CLgSSR46	MK959275	F:ACTCCCTTATTTCCATTTCTG	R:TGACCATTCATATAGGTCAGG	(CAT)7	Perfect	148
42	CLgSSR47	MK959276	F:GTTTATCAATTTGCCGAGTC	R:CAAGGAAGGAGGTCGATT	(AAC)6	Perfect	142
43	CLgSSR48	MK959277	F:TTGTTGAATATGTGTGGTCCT	R:GAAGAGGAGGATGAGCAAG	(CTC)6	Perfect	137
44	CLgSSR49	MK959278	F:CTCTACCATCTCCCTACCATT	R:GTGGAGGTGATTTGTAGATTG	(CTC)4	Perfect	150
45	CLgSSR50	MK959279	F:CATATGAGGAGAGAGGGATTC	R:GTTTGTACAACAGCGTCTCAC	(CAC)6	Perfect	150
46	CLgSSR51	MK959280	F:TCTCAATCTTGTGTGGATGAT	R:TTGGTCTTAATGTGGAAATTG	(AAT)6	Perfect	198
47	CLgSSR52	MK959281	F:ATGATCTTGTGAATGATCTCC	R:TTCAAGACACACAGGAGAAGT	(AAT)7	Perfect	131
48	CLgSSR53	MK959282	F:ACCACAACAATAGAGACAACG	R:TTACAGAGGCTGATTGTGAAT	(CAA)6	Perfect	148
49	CLgSSR54	MK959283	F:GGATAGCAAATCCATTTCATT	R:AAAGTTGGGATCAATAGTTGT	(ATC)6	Perfect	142
50	CLgSSR55	MK959284	F:GAGCATGCACAAATCTATGTC	R:TATCTTCTCATTAGGCACGTC	(AAG)6	Perfect	162
51	CLgSSR56	MK959285	F:AAACAATCTTGTGGATGATCT	R:CGTGCTACTTCAACAACTCTT	(ATA)6	Perfect	167
52	CLgSSR57	MK959286	F:ACAATAGCCCCAGTCATAGAT	R:CTACTTCAATCCTTGGTGATG	(CAA)7	Perfect	145
53	CLgSSR58	MK959287	F:TCAGTAGTAACGAGGATGAGC	R:TGATAAAGATTATCGCCTTTG	(GGC)6	Perfect	167
54	CLgSSR59	MK959288	F:AAGAAAGGAGAGTCATCAAGG	R:TGCATTAGGTTTCTTTAGAGG	(ACA)6	Perfect	152
55	CLgSSR60	MK959289	F:GTGCATTTACAATCAGAGAGC	R:TATTTGTACCGTTTCCATTGT	(CCA)6	Perfect	163
56	CLgSSR61	MK959290	F:CCAAAGTATGGAATGTTGTGT	R:CATATCCGGAGCAGTTTATAG	(AAT)6	Perfect	165
57	CLgSSR62	MK959291	F:GAACTGCTGCATTGATCC	R:GGTGGTGCAGTTCCAGAT	(CCT)6	Perfect	166
58	CLgSSR63	MK959292	F:TATAAATTACCGCAATCTTGG	R:CGGCTTACTTTATGTGAGTCT	(AAT)6	Perfect	116
59	CLgSSR64	MK959293	F:AGGAGATTGAGAAGAGAATGG	R:CACTCGATGTCAAGCTCTAAG	(GAG)6	Perfect	154
60	CLgSSR65	MK959294	F:GTGAAGCTCAATGTTTACACC	R:CACCTATCTTCAATCAGATGC	(CAT)6	Perfect	157
61	CLgSSR66	MK959295	F:AGACCTCTAAGGAGGAGAAGG	R:AGACATACCACTTGCAGTCAC	(GAG)7	Perfect	176
62	CLgSSR67	MK959296	F:CTCATACCAACCTTGAATTTG	R:AGCCATTGGGTTAATACTGAT	(TGA)7	Perfect	137
63	CLgSSR68	MK959297	F:CAAGAGGACAGACAGTGAGAG	R:TAGGTATTGGGTCAGTTGAAG	(GAG)6	Perfect	163
64	CLgSSR69	MK959298	F:ATAGTTTGGAAGTTGGGAATC	R:CCTAATAATAGGATGGGAACG	(TAC)7	Perfect	151
65	CLgSSR70	MK959299	F:TCCAAGAAGTATTGGATGATT	R:AATTTATGTTGAAGGTGCAAG	(ATG)6	Perfect	147
66	CLgSSR71	MK959300	F:ATATCAAGACGGATGAAACCT	R:CTTCCTCTGATTCGTATGTTG	(AGA)6	Perfect	153
67	CLgSSR72	MK959301	F:CTAGCTTCACTGACTCTCGTG	R:CATCAGAAGCATTCGAATAAG	(CTC)6	Perfect	148

continued the table

68	CLgSSR73	MK959302	F:CCTAGTAGGAGATTCGAGACC	R:TATGTCTCTCCTTCGTAGTGC	(GAG)6	Perfect	145
69	CLgSSR74	MK959303	F:AAGAGAAAATACGGAAACCTG	R:ACACATTTGGACAGAAAGATG	(ATC)6	Perfect	146
70	CLgSSR75	MK959304	F:CACCTTATTCCAACTTGCTAA	R:TGTTGAGAGATTTGATTTTGAG	(AC)6	Perfect	140
71	CLgSSR76	MK959305	F:AAAGTGCCTTTGTGTTGC	R:GAACAAGTAGAGGCTCAACAA	(TG)10	Perfect	150
72	CLgSSR77	MK959306	F:GTTTGCCTACATCCAATTCTA	R:CACAAACAAACTTCCATCAAT	(AT)6	Perfect	152
73	CLgSSR78	MK959307	F:GACATAATTGGTGCAACAGAG	R:CTTCTCTATCGTCAACACTGG	(AT)8	Perfect	151
74	CLgSSR79	MK959308	F:AGTAACCTCTGCTTCTTCCAG	R:TCATCAGCCTTATTGAGGATA	(TA)6	Perfect	149
75	CLgSSR80	MK959309	F:ATACATGTTCCATGGAGTTTC	R:GGGTGAGTTCAAGCAAGTC	(CA)7	Perfect	161
76	CLgSSR85	MK959310	F:TTTGCATTCAATAGTGGAAAC	R:TGACCTCTAGACCTCATTTAGC	(TG)6	Perfect	180
77	CLgSSR87	MK959311	F:AGGGCATCTGTTATTAGCTTC	R:ACAAAACAATTAAGACATCTGC	(GT)6	Perfect	142
78	CLgSSR88	MK959312	F:GTAACAGGGTCAAAATCAAGA	R:CACACCAAAGAAATGATAGGA	(TA)6	Perfect	163
79	CLgSSR89	MK959313	F:CCCTCTTCACACTTATTAGTCC	R:TGAATGATAAGGAACAAGGAT	(AT)6	Perfect	154

Table S3 Geographical origins, Q values and clustering results of different Chinese fir clones.

Clone name	Geographical provenance	Q1	Q2	Q3	Grouping based on STRUCTURE analysis	Grouping based on NJ-tree
Cl 1	Guangxi Rongshui	0.305	0.684	0.011	II	II
Cl 3	Guangxi Sanjiang	0.613	0.372	0.015	I	II
Cl 4	Guangxi Rongshui	0.501	0.474	0.025	Mix	II
Cl 5	Guangxi Rongshui	0.718	0.252	0.030	I	II
Cl 6	Guangxi Rongshui	0.304	0.659	0.037	II	II
Cl 7	Guangxi Rongshui	0.166	0.793	0.041	II	II
Cl 8	Guangxi Sanjiang	0.222	0.489	0.290	Mix	II
Cl 10	Guangxi Rongshui	0.567	0.405	0.028	Mix	II
Cl 11	Guangxi Rongshui	0.344	0.637	0.019	II	II
Cl 12	Guangxi Rongshui	0.503	0.412	0.085	Mix	II
Cl 13	Guangxi Rongshui	0.799	0.041	0.160	I	II
Cl 14	Guangxi Rongshui	0.775	0.075	0.150	I	III
Cl 15	Guangxi Rongshui	0.709	0.105	0.186	I	II
Cl 16	Guangxi Rongshui	0.648	0.303	0.050	I	III
Cl 17	Guangxi Rongshui	0.692	0.024	0.284	I	II
Cl 18	Guangxi Rongshui	0.931	0.033	0.036	I	I
Cl 19	Guangxi Rongshui	0.697	0.044	0.259	I	II
Cl 21	Guangxi Rongshui	0.792	0.201	0.006	I	II
Cl 24	Guangxi Rongshui	0.835	0.032	0.133	I	II
Cl 25	Guangxi Rongshui	0.985	0.010	0.005	I	I
Cl 26	Guangxi Sanjiang	0.994	0.003	0.003	I	I
Cl 27	Guangxi Rongshui	0.995	0.002	0.003	I	I
Cl 28	Guangxi Rongshui	0.993	0.003	0.004	I	I
Cl 29	Guangxi Rongshui	0.989	0.008	0.003	I	I
Cl 30	Guangxi Rongshui	0.972	0.025	0.003	I	I
Cl 31	Guangxi Rongshui	0.994	0.003	0.003	I	I
Cl 32	Guangxi Rongshui	0.989	0.005	0.005	I	I
Cl 33	Guangxi Rongshui	0.961	0.010	0.030	I	I
Cl 34	Guangxi Rongshui	0.993	0.004	0.004	I	I
Cl 35	Guangxi Rongshui	0.995	0.002	0.003	I	I
Cl 37	Guangxi Rongshui	0.988	0.002	0.010	I	I
Cl 38	Guangxi Rongshui	0.994	0.003	0.003	I	I
Cl 39	Guangxi Rongshui	0.987	0.008	0.006	I	I
Cl 40	Guangxi Rongshui	0.961	0.030	0.008	I	I
Cl 41	Guangxi Rongshui	0.988	0.006	0.006	I	I
Cl 42	Guangxi Rongshui	0.981	0.015	0.004	I	I
Cl 43	Guangxi Rongshui	0.993	0.002	0.004	I	I
Cl 44	Guangxi Rongshui	0.990	0.003	0.007	I	I
Cl 45	Guangxi Rongshui	0.980	0.007	0.013	I	I
Cl 46	Guangxi Nandan	0.983	0.012	0.005	I	I
Cl 47	Guangxi Nandan	0.811	0.005	0.185	I	I
Cl 48	Guangxi Nandan	0.987	0.010	0.004	I	I
Cl 50	Guangxi Liuzhou	0.984	0.003	0.013	I	I
Cl 51	Guangxi Liuzhou	0.991	0.005	0.004	I	I
Cl 52	Guangxi Liuzhou	0.993	0.003	0.004	I	I
Cl 53	Guangxi Napo	0.983	0.002	0.015	I	I
Cl 54	Guangxi Napo	0.995	0.002	0.003	I	I
Cl 55	Guangxi Napo	0.992	0.004	0.004	I	I
Cl 56	Guangxi Napo	0.988	0.006	0.006	I	I
Cl 57	Guangxi Napo	0.995	0.002	0.003	I	I
Cl 58	Guangxi Napo	0.994	0.003	0.004	I	I
Cl 59	Guangxi Napo	0.971	0.005	0.024	I	I
Cl 60	Guangxi Napo	0.991	0.003	0.006	I	I
Cl 61	Guangxi Napo	0.989	0.007	0.004	I	I
Cl 62	Guangxi Napo	0.993	0.003	0.004	I	I

continued the table

Cl 63	Hunan Jinxian	0.992	0.004	0.004	I	I
Cl 64	Hunan Jinxian	0.993	0.003	0.004	I	I
Cl 65	Hunan Jinxian	0.993	0.004	0.004	I	I
Cl 66	Hunan Jinxian	0.994	0.003	0.003	I	I
Cl 67	Hunan Jinxian	0.993	0.002	0.005	I	I
Cl 68	Hunan Jinxian	0.993	0.003	0.004	I	I
Cl 69	Hunan Jinxian	0.583	0.019	0.398	Mix	III
Cl 70	Hunan Jinxian	0.993	0.003	0.004	I	I
Cl 71	Hunan Jinxian	0.991	0.003	0.006	I	I
Cl 72	Hunan Jinxian	0.991	0.003	0.006	I	I
Cl 73	Hunan Huitong	0.993	0.003	0.004	I	I
Cl 74	Hunan Huitong	0.994	0.003	0.003	I	I
Cl 75	Hunan Huitong	0.987	0.010	0.003	I	I
Cl 76	Hunan Huitong	0.984	0.003	0.012	I	I
Cl 77	Hunan Huitong	0.989	0.003	0.008	I	I
Cl 78	Hunan Huitong	0.915	0.080	0.005	I	I
Cl 79	Hunan Huitong	0.995	0.002	0.003	I	I
Cl 80	Hunan Jinxian	0.995	0.002	0.003	I	I
Cl 81	Hunan Jinxian	0.993	0.003	0.004	I	I
Cl 82	Hunan Jinxian	0.992	0.004	0.005	I	I
Cl 83	Hunan Jinxian	0.992	0.003	0.005	I	I
Cl 84	Hunan Jinxian	0.992	0.004	0.005	I	I
Cl 85	Hunan Huitong	0.709	0.108	0.183	I	I
Cl 86	Hunan Lingling	0.811	0.173	0.016	I	I
Cl 87	Hunan Jinxian	0.979	0.007	0.014	I	I
Cl 88	Hunan Jinxian	0.802	0.143	0.054	I	I
Cl 89	Hunan Jinxian	0.706	0.220	0.074	I	I
Cl 90	Hunan Jinxian	0.920	0.031	0.049	I	I
Cl 91	Hunan Jinxian	0.842	0.076	0.083	I	I
Cl 92	Hunan Jinxian	0.984	0.005	0.011	I	I
Cl 93	Guizhou Jinping	0.864	0.130	0.006	I	I
Cl 94	Guizhou Jinping	0.802	0.186	0.012	I	I
Cl 95	Guizhou Jinping	0.938	0.018	0.044	I	I
Cl 96	Guizhou Jinping	0.913	0.047	0.041	I	I
Cl 97	Guizhou Jinping	0.004	0.211	0.786	III	III
Cl 98	Guizhou Jinping	0.007	0.015	0.978	III	III
Cl 99	Guizhou Jinping	0.017	0.260	0.723	III	III
Cl 100	Guizhou Jinping	0.005	0.085	0.911	III	III
Cl 101	Guizhou Jinping	0.014	0.153	0.834	III	III
Cl 102	Guizhou Jinping	0.004	0.370	0.626	III	III
Cl 103	Guizhou Jinping	0.090	0.050	0.861	III	III
Cl 104	Guizhou Jinping	0.015	0.489	0.496	Mix	III
Cl 105	Guizhou Jinping	0.050	0.453	0.497	Mix	III
Cl 106	Guizhou Tianzhu	0.004	0.390	0.605	III	III
Cl 107	Guizhou Tianzhu	0.012	0.137	0.851	III	III
Cl 108	Guizhou Tianzhu	0.003	0.275	0.722	III	III
Cl 109	Guizhou Tianzhu	0.027	0.035	0.938	III	III
Cl 110	Guizhou Liping	0.021	0.059	0.920	III	III
Cl 111	Guizhou Liping	0.011	0.005	0.985	III	III
Cl 112	Guizhou Liping	0.006	0.051	0.943	III	III
Cl 113	Guangxi Rongshui	0.005	0.003	0.992	III	III
Cl 114	Guangxi Rongshui	0.012	0.007	0.981	III	III
Cl 115	Guangxi Rongshui	0.004	0.004	0.993	III	III
Cl 117	Guangxi Rongshui	0.004	0.082	0.914	III	III
Cl 118	Guangxi Rongshui	0.005	0.134	0.861	III	III
Cl 119	Guangxi Rongshui	0.004	0.053	0.943	III	III

continued the table

Cl 120	Guangxi Rongshui	0.006	0.017	0.978	III	III
Cl 122	Guangxi Rongshui	0.004	0.005	0.991	III	III
Cl 123	Guangxi Rongshui	0.004	0.002	0.994	III	III
Cl 124	Guangxi Rongshui	0.006	0.009	0.986	III	III
Cl 125	Guangxi Rongshui	0.005	0.003	0.992	III	III
Cl 127	Guangxi Rongshui	0.003	0.003	0.994	III	III
Cl 129	Guangxi Rongshui	0.003	0.003	0.994	III	III
Cl 130	Guangxi Sanjiang	0.003	0.003	0.994	III	III
Cl 131	Guangxi Sanjiang	0.004	0.006	0.990	III	III
Cl 132	Guangxi Sanjiang	0.009	0.015	0.976	III	III
Cl 133	Guangxi Sanjiang	0.100	0.006	0.894	III	III
Cl 134	Guangxi Sanjiang	0.004	0.005	0.991	III	III
Cl 135	Guangxi Sanjiang	0.004	0.002	0.994	III	III
Cl 136	Guangxi Sanjiang	0.005	0.003	0.992	III	III
Cl 137	Guangxi Sanjiang	0.005	0.005	0.990	III	III
Cl 138	Guangxi Sanjiang	0.101	0.005	0.894	III	III
Cl 139	Guangxi Sanjiang	0.005	0.005	0.991	III	III
Cl 140	Guangxi Sanjiang	0.005	0.007	0.988	III	III
Cl 141	Guangxi Sanjiang	0.002	0.003	0.995	III	III
Cl 142	Guangxi Sanjiang	0.005	0.004	0.992	III	III
Cl 143	Guangxi Sanjiang	0.003	0.007	0.990	III	III
Cl 144	Guangxi Sanjiang	0.006	0.009	0.985	III	III
Cl 145	Guangxi Sanjiang	0.007	0.025	0.968	III	III
Cl 146	Guangxi Sanjiang	0.009	0.004	0.987	III	III
Cl 147	Guangxi Sanjiang	0.003	0.005	0.992	III	III
Cl 148	Guangxi Rongshui	0.006	0.010	0.984	III	III
Cl 149	Guangxi Rongshui	0.003	0.019	0.978	III	III
Cl 150	Guangxi Rongshui	0.002	0.004	0.994	III	III
Cl 151	Guangxi Rongshui	0.003	0.004	0.993	III	III
Cl 152	Guangxi Rongshui	0.003	0.003	0.994	III	III
Cl 153	Guangxi Rongshui	0.004	0.003	0.993	III	III
Cl 154	Guangxi Rongshui	0.003	0.002	0.995	III	III
Cl 155	Guangxi Rongshui	0.003	0.003	0.995	III	III
Cl 156	Guangxi Rongshui	0.004	0.003	0.993	III	III
Cl 157	Guangxi Rongshui	0.024	0.007	0.969	III	III
Cl 159	Guangxi Rongshui	0.006	0.008	0.987	III	III
Cl 160	Guangxi Rongshui	0.007	0.005	0.988	III	III
Cl 162	Guangxi Rongshui	0.008	0.006	0.986	III	III
Cl 164	Guangxi Rongshui	0.028	0.013	0.959	III	III
Cl 165	Guangxi Rongshui	0.005	0.004	0.991	III	III
Cl 166	Guangxi Rongshui	0.004	0.003	0.993	III	III
Cl 167	Guangxi Rongshui	0.005	0.002	0.993	III	III
Cl 168	Guangxi Rongshui	0.003	0.005	0.992	III	III
Cl 169	Guangxi Rongshui	0.029	0.003	0.968	III	III
Cl 170	Guangxi Rongshui	0.004	0.010	0.987	III	III
Cl 172	Guangxi Rongshui	0.004	0.003	0.993	III	III
Cl 173	Guangxi Rongshui	0.012	0.005	0.983	III	III
Cl 175	Guangxi Rongshui	0.006	0.003	0.991	III	III
Cl 177	Guangxi Rongshui	0.016	0.005	0.979	III	III
Cl 178	Guangxi Rongshui	0.005	0.003	0.992	III	III
Cl 179	Guangxi Rongshui	0.003	0.002	0.995	III	III
Cl 180	Guangxi Rongshui	0.005	0.004	0.991	III	III
Cl 182	Guangxi Rongshui	0.082	0.002	0.916	III	III
Cl 184	Guangxi Rongshui	0.004	0.003	0.993	III	III

continued the table

Cl 187	Guangxi Rongshui	0.067	0.014	0.919	III	III
Cl 188	Guangxi Rongshui	0.017	0.074	0.908	III	III
Cl 189	Guangxi Rongshui	0.005	0.004	0.991	III	III
Cl 190	Guangxi Rongshui	0.008	0.016	0.976	III	III
Cl 191	Guangxi Rongshui	0.004	0.003	0.993	III	III
Cl 195	Guangxi Rongshui	0.305	0.685	0.011	II	II
Cl 197	Guangxi Rongshui	0.003	0.995	0.002	II	II
Cl 198	Guangxi Rongshui	0.004	0.992	0.005	II	II
Cl 200	Guangxi Rongshui	0.003	0.995	0.002	II	II
Cl 201	Guangxi Sanjiang	0.014	0.974	0.012	II	II
Cl 202	Guangxi Sanjiang	0.004	0.993	0.004	II	II
Cl 203	Guangxi Sanjiang	0.038	0.959	0.003	II	II
Cl 204	Guangxi Napo	0.091	0.903	0.006	II	II
Cl 205	Guangxi Napo	0.009	0.919	0.072	II	II
Cl 206	Guangxi Napo	0.012	0.959	0.029	II	II
Cl 207	Guangxi Napo	0.016	0.955	0.030	II	II
Cl 208	Guangxi Napo	0.003	0.989	0.008	II	II
Cl 209	Guangxi Hechi	0.012	0.980	0.008	II	II
Cl 210	Guangxi Hechi	0.003	0.994	0.003	II	II
Cl 211	Guangxi Hechi	0.003	0.995	0.002	II	II
Cl 212	Guangxi Hechi	0.004	0.993	0.003	II	II
Cl 214	Guangxi Hechi	0.005	0.990	0.005	II	II
Cl 215	Guangxi Hechi	0.004	0.991	0.005	II	II
Cl 216	Guangxi Hechi	0.003	0.995	0.002	II	II
Cl 217	Guangxi Hechi	0.013	0.982	0.005	II	II
Cl 218	Guangxi Hechi	0.007	0.989	0.004	II	II
K 0	Zhejiang Kaihua	0.440	0.006	0.555	Mix	III
K 36	Zhejiang Kaihua	0.586	0.004	0.410	Mix	III
K 40	Zhejiang Kaihua	0.562	0.023	0.415	Mix	III
K 78	Zhejiang Kaihua	0.527	0.018	0.456	Mix	III
K 86	Zhejiang Kaihua	0.518	0.010	0.473	Mix	III
L 317	Zhejiang Linan	0.534	0.010	0.456	Mix	III
L 15	Zhejiang Longquan	0.599	0.007	0.394	Mix	III
M 33	Fujian	0.449	0.133	0.418	Mix	III
